# The puzzling mitochondrial phylogeography of the black soldier fly (*Hermetia illucens*), the commercially most important insect protein species

**DOI:** 10.1186/s12862-020-01627-2

**Published:** 2020-05-24

**Authors:** Gunilla Ståhls, Rudolf Meier, Christoph Sandrock, Martin Hauser, Ljiljana Šašić Zorić, Elina Laiho, Andrea Aracil, Jovana Doderović, Rozane Badenhorst, Phira Unadirekkul, Nur Arina Binte Mohd Adom, Leo Wein, Cameron Richards, Jeffery K. Tomberlin, Santos Rojo, Sanja Veselić, Tuure Parviainen

**Affiliations:** 1 Finnish Museum of Natural History Luomus, Zoology unit, University of Helsinki, PO Box 17, FI-00014 Helsinki, Finland; 2 Department of Biological Sciences, National University of Singapore, Block S3 #05-01, 14 Science Dr 4, Singapore, 117543 Singapore; 3 Department of Livestock Sciences, Research Institute of Organic Agriculture (FiBL), Ackerstrasse 113, 5070 Frick, Switzerland; 4 California Department of Food and Agriculture, Plant Pest Diagnostics Branch, 3294 Meadowview Road, Sacramento, California 95832-1448 USA; 5 BioSense Institute, Dr Zorana Đinđića 1, Novi Sad, 21000 Serbia; 6 Department of Environmental Sciences & Natural Resources, University of Alicante, PO Box 99, E-03080 Alicante, Spain; 7 Department of Biology and Ecology, Faculty of Sciences, University of Novi Sad, Novi Sad, 2100 Serbia; 8 Agriprotein, 1 Rochester Road, Cape Town, South Africa; 9 Protenga, 302 Ang Mo Kio Ave 3, #01-1840 560302, Singapore, Singapore; 10 Department of Entomology, Texas A&M University, TAMU 2475, College Station, TX 77843-2475 USA; 11 VTT Technical Research Centre of Finland Ltd, TT2 Tietotie 2, P.O. Box 1000, FI-02044 VTT Espoo, Finland

**Keywords:** Black soldier fly, *Hermetia illucens*, mtDNA COI haplotypes, Nuclear rDNA

## Abstract

**Background:**

The black soldier fly (Diptera: Stratiomyidae, *Hermetia illucens*) is renowned for its bioconversion ability of organic matter, and is the worldwide most widely used source of insect protein. Despite varying extensively in morphology, it is widely assumed that all black soldier flies belong to the same species, *Hermetia illucens*. We here screened about 600 field-collected and cultured flies from 39 countries and six biogeographic regions to test this assumption based on data for three genes (mitochondrial COI, nuclear ITS2 & 28S rDNA) and in order to gain insights into the phylogeography of the species.

**Results:**

Our study reveals a surprisingly high level of intraspecific genetic diversity for the mitochondrial barcoding gene COI (divergences up to 4.9%). This level of variability is often associated with the presence of multiple species, but tested nuclear markers (ITS2 and 28S rDNA) were invariant and fly strain hybridization experiments under laboratory conditions revealed reproductive compatibility. COI haplotype diversity is not only very high in all biogeographic regions (56 distinct haplotypes in total), but also in breeding facilities and research centers from six continents (10 haplotypes: divergences up to 4.3%). The high genetic diversity in fly-breeding facilities is mostly likely due to many independent acquisitions of cultures via sharing and/or establishing new colonies from field-collected flies. However, explaining some of the observed diversity in several biogeographic regions is difficult given that the origin of the species is considered to be New World (32 distinct haplotypes) and one would expect severely reduced genetic diversity in the putatively non-native populations in the remaining biogeographic regions. However, distinct, private haplotypes are known from the Australasian (*N* = 1), Oriental (*N* = 4), and the Eastern Palearctic (N = 4) populations. We reviewed museum specimen records and conclude that the evidence for introductions is strong for the Western Palearctic and Afrotropical regions which lack distinct, private haplotypes.

**Conclusions:**

Based on the results of this paper, we urge the black soldier fly community to apply molecular characterization (genotyping) of the fly strains used in artificial fly-breeding and share these data in research publications as well as when sharing cultures. In addition, fast-evolving nuclear markers should be used to reconstruct the recent invasion history of the species.

## Background

Black Soldier Flies (BSF), *Hermetia illucens* (Linnaeus, 1758) (Diptera, Stratiomyidae) are large flies with brownish wings and a pair of characteristic translucent pale patches (windows) on the abdomen [1]. The larvae of this synanthropic species are very effective decomposers of an exceptionally wide range of decaying organic matter including manure, food waste, agricultural by-products, organic leachates and cadavers (including human) [2–5]. Not surprisingly, the BSF has thus emerged as the dominant source of insect protein and is now utilized at a large scale worldwide due to its ability to convert organic matter into oil- and protein-rich feed (for a recent review see [6]). Indeed, the commercial value of BSF larvae is considered so significant that several types of larvae have registered trademark names: Phoenix Worms®, CalciWorms®, ReptiWorms™ and CalciGrubs (see https://phoenixworm.com/) [7]. In nature, adult flies rarely feed but may occasionally imbibe water or honeydew. Therefore, the larvae need to accumulate enough protein and fat during the six larval stages in order to provision the females with sufficient resources for producing 500–1000 eggs [8] and males with sufficient energy for sperm production and mating on the wing. Commercial producers in Africa, Asia, Europe, North and South America presently rear large numbers of larvae that are used as feed in aquaculture and poultry farms [9, 10]. Many cultures were started using the immature stages of commercially available Phoenix Worms®, which have been supplied for 20 years [3]. However, the origin for many new rearing cultures is unknown. This is problematic because it is common for cultures to be shared without any prior genetic characterization (e.g. genotyping).

The native range of BSF is considered to be New World, from northern parts of South America to the northern-most native populations occurring in the southwestern parts of the US (at least up to 40° N) [11]. However, the species is now cosmopolitan and occurs in all other tropical, as well as many subtropical and temperate regions (between 46°N and 40°S) [11–13]. All the Old World populations are considered introduced. Indeed, numerous historical records from the late 19th and early twentieth-century document the presence of the species in the New World [13] while the earliest records for the other regions are considerably younger. For the Afrotropical region, it is a South African record from 1915 [13] and several subsequent ones from Madagascar in the 1930s [14]. The first record for the Palaearctic region is from Malta in 1926 [15], but by the 1960s the species was known from Spain, France and Italy [12, 16]. In Asia the first confirmed records are from the 1940s (e.g., Malaysia) but there are no confirmed records from, for example, China until 1960 [13]. With regard to the Australasian region, reports of *H. illucens* specimens from Australia collected in 1915 [16, 17] have now been revealed to be erroneous because they belong to a related species (*H. pallidipes* Hill, 1919) [18]. The oldest known specimen for the conspicuous *H. illucens* is thus from 1948 (in Australian National Insect Collection). According to Marshall et al. [13] many Pacific islands were colonized by *H. illucens* by the1940s. This includes Hawaii, the Solomon Islands, New Caledonia, Mariana Islands, Palau, and Guam. Overall, it is striking that many of the earliest known localities outside of the New World are on islands or close to the coast. This may indicate that worldwide trade played a major role in the passive spread of BSF [12]. For example, introduction via shipping is likely responsible for the fast spread of the species throughout the Pacific region in the 1940s (e.g., Guam and Palau) given that the species’ arrival coincided with troop and supply movements during WWII.

Species with exceptionally large distributions are frequently revealed to consist of species complexes or comprise several evolutionary significant units that deserve recognition once they are studied using systematic investigation using genetic methods in order to protect their genetic integrity [19–21]. Fragmentary evidence has been emerging that BSF may be such a species. For example, a regional study of mitochondrial cytochrome c oxidase I (COI) barcode variability in South Korea [22] revealed 10 highly diverged haplotypes. If confirmed for the populations and cultures worldwide, such high genetic diversity would have considerable implications for scientific research and the commercial use of the species. For example, it took decades until genotyping revealed the presence of several species in widely used experimental cultures of leeches in the genus *Helobdella*. By the time of discovery, the lack of timely genotyping had done severe harm to the scientific literature because results had been filed under incorrect scientific names [23]. Shortly after, similar problems were discovered for a “medical device”, the medicinal leech *Hirudo medicinalis*. Many leeches used for medical purposes were revealed to belong to species that were not approved for use [24]. Yet, BSF captive cultures used in science or by companies have not been genotyped and very little is known about the genetic diversity of the species worldwide.

We here carry out a first systematic survey across the species’ current range with the aim toreconstruct the phylogeography of the target species. This is particularly challenging because the species has apparently been introduced repeatedly due to international trade in many regions over the last decades. Additional complication stem from the species’ being prone to escape from rearing cultures, and the international trade by commercial breeders (e.g. providing online ordering utilized by e.g. exotic-pet keepers). We therefore here compare the genetic diversity of field-collected flies (of which some, however, could be quite recent escapees) with samples obtained from rearing cultures maintained for academic or commercial purposes. In addition to mitochondrial data, we assess the genetic diversity for two fast-evolving nuclear markers (ITS2 and a loop region of 28S rDNA [25–27];. Lastly, we test whether two cultures with high genetic divergence for COI are reproductively compatible.

## Results

### COI barcode dataset and analyses

**Fig. 1:**
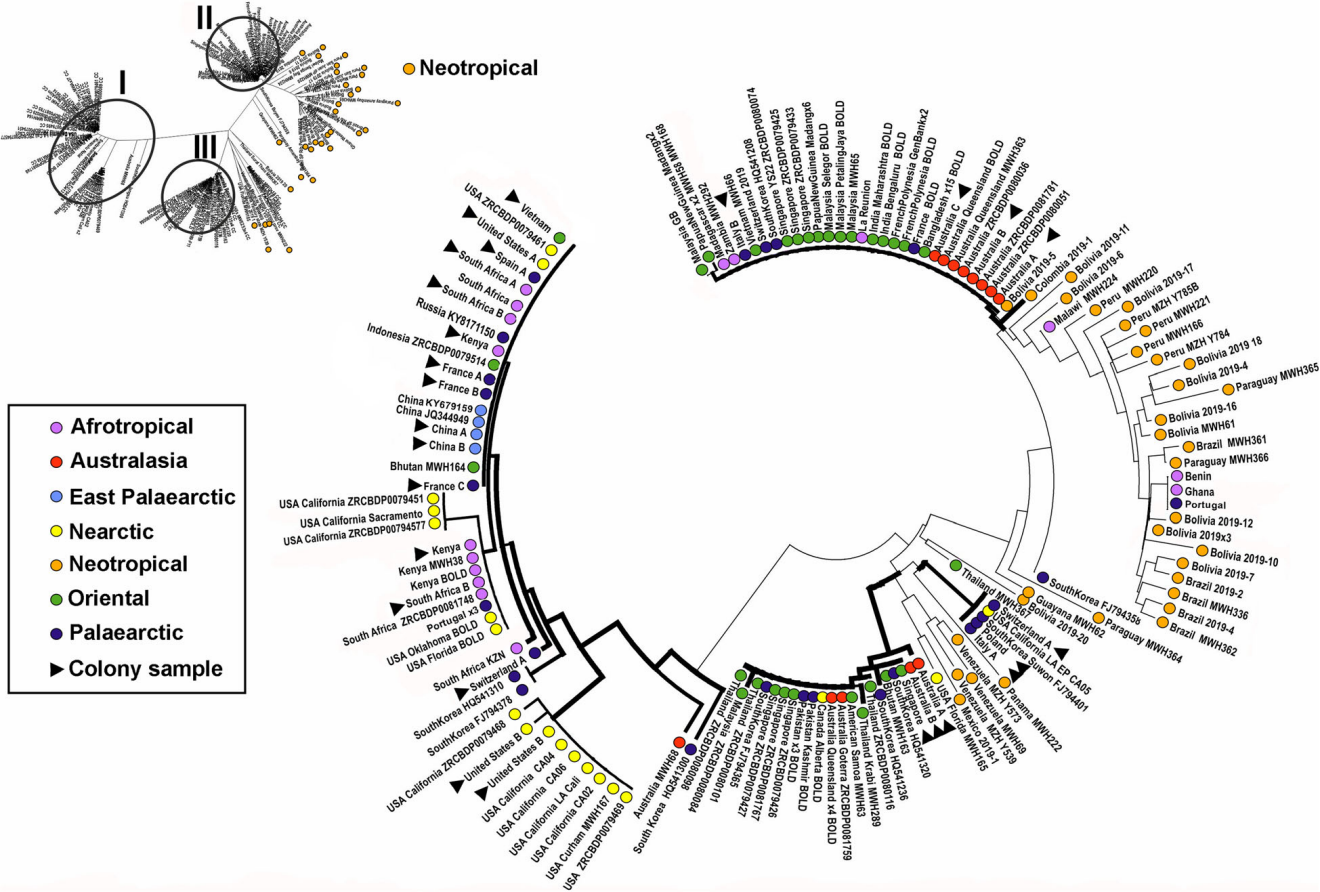
Neighbor-Joining network (uncorrected p-distances) of all haplotypes (labelled with country and sample code). The biogeographical region of the haplotypes is indicated with color-coded filled circles (purple = Afrotropical, pink = Australasia, green = Oriental, orange = Neotropical, yellow = Nearctic, dark blue = Palaearctic). All rearing culture samples are indicated with a black ►. The most abundant COI haplotype resolved in sequence clusters are indicated with Roman numerals I-III with the geographical sources listed in Table 1

**Fig. 2:**
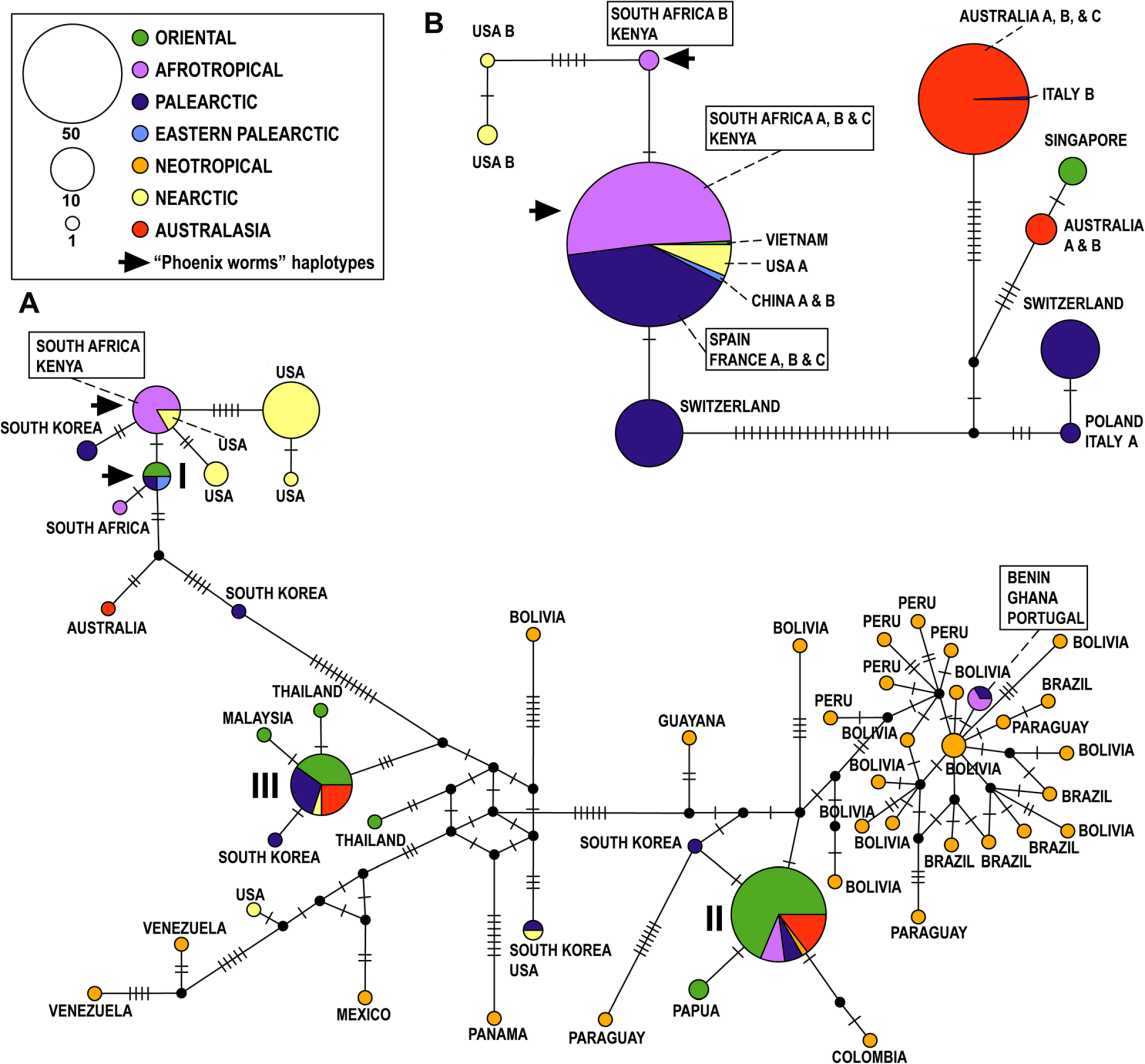
**a.** Median-Joining haplotype network of all field-collected COI haplotypes. The black circles represent putative un-sampled haplotypes. The branch lengths are largely proportional to the numbers of mutational steps separating the haplotypes, and the number(s) of mutational steps are indicated with hashmarks on the branches. The biogeographical region of the haplotypes is color-coded, and the size of circles is proportional to the number of individuals per haplotype. The most abundant COI haplotypes are indicated with Roman numerals I-III with the geographical sources listed in Table 1. **b.** Median-Joining network of the ten rearing culture COI haplotypes. Descriptions as in Fig. 2a

All COI barcodes were trimmed to equal lengths of 658 nt, and the dataset had no gaps. The A + T content was 61.7%, and 79/658 (12%) nucleotide sites were variable that coded for amino-acid changes in 5 of the 219 residues (2.3%). Our study screened > 500 fly rearing culture samples and barcoded 75 field-collected samples. These data were complemented with 49 publically available DNA barcodes from sequence databases which represented additional haplotypes and/or geographic localities (Supplementary data Table S1). All sequences were translatable and our repeated sequencing of the same specimens (15 samples representing all colonies and field-collected samples from several localities) yielded the same sequences for the same specimens. For subsequent analyses, identical COI barcodes from rearing culture samples and field-collected samples from a given geographic location were merged to retain only single representative barcodes of each haplotype (HT). The final COI dataset used for Neighbor-Joining network tree thus comprised 138 distinct barcodes representing 56 haplotypes, while the Median-Joining haplotype network had 135 barcodes representing 51 unique haplotypes (Supplementary Table S2; three incomplete sequences and four nucleotide positions excluded due to incomplete data in the latter dataset). We registered a total of 48 new haplotypes based on barcodes for the included samples and additionally 11 haplotypes already registered in public databases*.* The maximum uncorrected p-distance across the full dataset was 4.9%. The NJ-network tree of all COI barcodes was resolved three larger sequence clusters (Fig. 1: I-III), each comprising both cultured and wild flies, while Neotropical samples mainly grouped in one larger and one smaller sequence cluster (Fig. 1). The Median-Joining haplotype network for the dataset of field-collected flies resolved the same sequence clusters (Fig. 2a).

### COI haplotypes: non-cultured flies

We barcoded field-collected flies (56 sequenced in Helsinki lab, 21 in Singapore) and added 49 barcodes from public databases (Supplementary Table S1). Field-collected flies from the Neotropics show the highest number of distinct, private haplotypes (32 out of a total of 56) mainly represented by single individuals with sequence divergences up to 3.7% among them. Several unique haplotypes were recorded for the Australasian (1 haplotype), Oriental (4) and Palaearctic (4) samples (Figs. 1; 2a). For single samples from the Afrotropical and Palaearctic regions (Benin, Ghana, Malawi and Portugal) we found a haplotype nested among the New World haplotypes (Figs. 1; 2a). Samples from La Reunion, Madagascar, and Zambia (Afrotropical) shared haplotypes with samples from the Australasian, Neotropical, Oriental and Palaearctic and regions (Fig. 1: II; Table 1). A haplotype recorded for the Australasian and Oriental regions was also found in samples from the Nearctic and Palaearctic (Fig. 1: III; Table 1).

**Table 1:** Occurrences of partly shared haplotypes between rearing cultures and field-collected flies and resolved into three large sequence clusters (Fig. 1 I-III; Fig. 2a). In culture: Country of origin of the rearing facilities (different facilities in the same country indicated with letters A-C) of the studied BSF cultures. The total number of sequenced samples for culture samples in each country indicated in brackets. In nature: Origin of samples obtained from both pristine habitats and other natural areas (sometimes close to fly rearing facilities) with haplotypes identical to cultured flies

	In culture	In nature
**I**	China: A & B (2); France: A, B & C (3); Kenya (17); South Africa: A, B & C (57); Spain (55); Switzerland (24); USA: A & B (9); Vietnam (1).	AFROTROPICAL: Kenya, South Africa; NEARCTIC: USA; ORIENTAL: Bhutan, China, Indonesia; PALAEARCTIC: Portugal, Russia, South Korea; EASTERN PALAEARCTIC: China.
**II**	Australia A, B & C (65); Italy B (1).	AFROTROPICAL: La Reunion, Madagascar, Zambia; AUSTRALASIA: Australia; ORIENTAL: French Polynesia, India, Malaysia, Papua New Guinea, Singapore, Vietnam, Bangladesh; PALAEARCTIC: France, South Korea, Switzerland; NEOTROPICAL: Bolivia.
**III**	Australia A & B (5); Singapore (4).	AUSTRALASIA: Australia; PALAEARCTIC: Pakistan, South Korea; ORIENTAL: Singapore, Thailand, American Samoa, Bhutan, Malaysia; NEARCTIC: Canada.

### COI haplotypes: cultured flies

We sequenced the full-length COI barcode for altogether 292 samples (276 sequenced in Helsinki lab, 16 in Singapore) and recorded ten COI haplotypes from the rearing cultures (barcodes indicated with a black triangle in Figs. 1; 2b). Two shared, distinct haplotypes were recorded for samples that can be traced back to the commercial provider of ‘Phoenix worms’. These samples came from China, France, Kenya, South Africa, Spain, USA and Vietnam (Fig. 1: I; Fig. 2b). The same haplotypes were also found repeatedly in field-collected flies (of which some are probably escapees) in most of these countries (Table 1). In contrast, some commercial BSF providers in Australia rear ‘local’ flies with private, distinct haplotypes. A private haplotype was also recorded for samples from two rearing facilities in Europe (Fig. 2b), and one European rearing culture (Switzerland) had two distinct haplotypes. The most divergent rearing culture samples differed by p-distances of up to 4.1%.

### Nuclear ribosomal DNA

We obtained 453 nt of the ITS2 region for 196 samples, and only one nucleotide site was variable (some individuals were heterozygous), while all 28S loop sequences were invariant.

### Hybridization experiments

Mating trials under laboratory conditions revealed a lack of reproductive isolation between the populations, as hybrid offspring were produced and successfully back-crossed with the parental population. The hybrid populations were also fertile and some have now been maintained for > 10 generations. Backcrosses were not attempted for these.

## Discussion

### High COI barcode diversity

Our data reveal many unexpected results for this widely used workhorse of insect protein production. They highlight that there is currently a surprising lack of background information for a species that is globally, and extensively, used and shared for scientific research, animal feed production and organic waste recycling.

Our study revealed very high COI barcode variability, but this variability is not mirrored by the two invariant nuclear markers that were included in our study. This high COI diversity also does not translate into reproductive incompatibility although the divergence levels (close to 5%) well exceeds what is typical for flies [28]. This implies that the lack of genotyping of cultures may not have done as much scientific and commercial harm in BSF as it did for leeches belonging to *Helobdella* and *Hirudo*. The high COI diversity is structured in both expected and unexpected ways. The species is considered native to the New World; i.e., the high genetic diversity we recorded for the New World samples corresponds well with expectations, while the remaining regions should mostly have subsets of the New World genetic diversity. This is indeed the case for the Afrotropical and the Western Palearctic populations, which contain a limited number of haplotypes. It appears likely that these populations were indeed introduced. However, it was surprising to find several distinct, highly diverged haplotypes in Australasian, Oriental and Eastern Palaearctic populations, and this diversity remains unexplained if the species indeed originated in the New World and only recently dispersed to these regions. Possible explanations include still insufficient sampling of the Neotropical diversity or a broader geographic origin of the species.

BSF has not only unusually high levels of genetic diversity for COI, but it is also morphologically very variable with regard to body size, wing and abdominal coloration, and the shape of translucent abdominal windows. Wing coloration varies and ranges from almost black with a blueish-metallic shine over a clear brownish-opaque insignia in the inner part of the wing to almost pale-translucent. The amount of white versus black coloration on the head shows great variation, while the black and white pattern on the legs seem to be more consistent. There is a tendency for males to have a reddish to red abdomen, while females rarely display red coloration on the abdomen. Much of this variation can be found within the same laboratory cultures and is thus likely due to polyphenism, but more systematic study across cultures representing different haplotype groups is needed.

### Global trade and the distribution of BSF

We documented that the distinct haplotypes of the commercial US breeding strain (Figs. 1 and 2 a: the haplotypes of the commercial ‘Phoenix Worms’ indicated with arrows) can be found in flies from natural and urban environments in the Afrotropical (South Africa, Kenya), Oriental (Bhutan, China, Indonesia) and the Palaearctic (Portugal, Russia, Spain) regions, but it cannot be ascertained whether the flies originated from (repeated) introductions or if flies are escapees from rearing cultures. Similarly, haplotypes of flies recorded in commercial and/or research cultures in Asia and Australasia are also found in field-collected flies from these and other biogeographical regions (Fig. 1: II & III; Table 1). Barcodes of single flies sampled from Benin, Ghana, Malawi and Portugal were most similar to BSF barcodes from the Neotropical region (Figs. 1 and 2 a). These patterns are compatible with human-mediated introduction across biogeographic regions, but it will require population-level nuclear markers to confirm whether the introductions were sufficiently recent to be due to global trade.

Arguably, the most convincing evidence for human-mediated introductions come from specimens collected at latitudes that are unlikely to support breeding populations [13, 29–31]. The lower threshold for BSF larval survival was observed to be 15–19 °C depending on study [32, 33], and some Canadian BSF localities are unlikely to meet this requirement. Marshall et al. [13] thus hypothesized that the annual occurrence of BSF in Ontario (Canada) is due to the disposal of unused fishing baits (larvae) by local fishermen or the accidental release of flies into nature by owners of pet reptiles. However, the evidence is not entirely conclusive because BSF flies, prepupae, and pupae are resistant to cold and can survive low temperatures (5 °C) for several weeks [34]. This may explain why there is an established BSF population in Northwestern Switzerland that has been regularly encountered for over a decade. In addition, wild BSF are occasionally caught in Western and Central European countries such as Germany [29] and the Czech Republic [31]. Overall, more research is needed in order to fully understand the thermal tolerance of BSF and its diapausing abilities. This and all other research needs to be based on genotyped cultures because it is likely that populations from different parts of the (native) range will differ with regard to thermal tolerance. One way or another, it appears likely that BSF will be able to increase its current range as global warming takes hold. We would suggest that urban populations will most likely spearhead the range expansion because they benefit readily available organic waste and benefit from higher temperatures in urban environments [32]. Range expansion may also be facilitated through population homogenization as more cultures are shared over long distances and escaped flies cross-breed with native or previously established non-native populations.

The BSF is now being actively domesticated worldwide [35], but the field lacks ‘herdbook’ keeping as a means to track the fly strains (see international *Drosophila* stock centers). The scientific utility for the genetic characterization and source documentation in commercial breeding of domestic livestock (e.g. pigs and cattle) is obvious, and genomics has not only proven useful for reconstructing the domestication history of the different breeds, but also for understanding the molecular mechanisms underlying traits of interest [36]. A tremendous amount of phenotypic plasticity among three different BSF strains in the ability of their larvae to reduce dry matter and associated nutrients in three different animal manures was demonstrated in a study by Zhou et al. [7], and additional evidence is emerging more recently [37]. Knowing the link between genes and trait has improved target-based breeding of domestic livestock [38], and it seems obvious that it can also be achieved for ‘mini-livestock’ like the BSF. Linked characterization of both the nuclear and mitochondrial genomes could prove useful, as the mtDNA COI barcode could provide sufficient initial and inexpensive information for avoiding, for example potential reduced fitness resulting from the hybridization of highly diverged strains.

### Puzzling phylogeography

This study screened a comprehensive number of both cultured and wild caught BSF samples from six continents and 39 countries, including numerous from the putatively native New World and continents where BSF is an alien species. Our findings clearly highlight the need for additional sampling of New World populations as well as the Australasian and Oriental regions. In particular, the discovery of the haplotypes which are currently only known from Australasian and Oriental populations would strengthen the hypothesis of a New World origin of the species.

If a divergence rate of 2.3% per million years for insect mitochondrial DNA [39] were used as a guide, one would have to conclude that the most distant samples of the BSF haplotype network (Fig. 1) have been separated for 0.74–1.35 million years. This would imply that the Oriental and Australasian wild samples (Fig. 1: II & III) had already diverged 1.04 MY ago. These findings could either challenge the assumption of a New World origin or the spread of BSF was not human-mediated. Arguably, the strongest argument against the latter is that black soldier flies are conspicuous and should have been collected by early naturalists in Asia and Australasia if they had been present. Instead, the earliest records are from the 1940s.

## Conclusions

A high genetic diversity has considerable implications for scientific research and commercial use of the species. Assessing the genetic makeup of cultures would seem a necessary fly culture management tool, considering the possible advantages of a high genetic diversity of the BSF strains coupled with different dominant characteristics that a breeding program could capitalize on. We therefore urge all commercial users and researchers to generate barcodes for their cultures given that this is low cost. The DNA and specimens should also be kept for future screening of additional nuclear markers. Based on our results, the mtDNA COI barcode is sufficiently informative to be a first step marker for any genetic characterization of BSF strains or populations. The available genomic studies on the BSF are still limited [40, 41], but are expected to rapidly increase in near future, and are likely to yield additional markers. Finally, in order to unravel a more detailed dispersal history of the BSF, comprehensive population genetic analyses should be carried out.

## Methods

Our study included both samples from cultures and the wild. For cultures, we screened multiple individuals (adult flies or larvae) from rearing facilities in Europe, Africa, Asia, Australia and United States. Field-collected (“wild”) fly samples were collected in both urban and pristine habitats from Africa, Asia, Australasia, Europe, and the Americas, but it is often difficult to rule out that they are escaped flies from cultures. Our data were complemented with publicly available COI barcodes retrieved from GenBank (ncbi.nih.gov) and BOLD (boldsystems.org) (for data on BSF samples, GenBank accession codes and repositories see Supplementary Table S1).

### Laboratory procedures

Helsinki lab. *Cultured samples*: Genomic DNA was extracted from 276 samples from four countries. About half of the thorax of an individual ethanol preserved fly, or from a small piece (2 × 2 mm) of larval tissue was used for DNA extraction with the Nucleospin Tissue DNA extraction kit (Macherey-Nagel, Düren, Germany). Ready-to-Go PCR beads (GE Healthcare, Little Chalfont, UK) were used for amplification of the COI barcode using the universally conserved primer pairs LCO-1490 and HCO-2198 [42] of all samples.

*Field-collected samples*: Fly specimens were obtained from field collections or from the authors’ conserved collections (Supplementary Table S1). Genomic DNA was extracted using 1–2 legs of each pinned fly specimen, as a least-invasive approach. The Phire™ Tissue Direct PCR Master Mix #F-170S kit (Thermo Scientific Baltics UAB, Vilnius, Lithuania) was used for DNA extraction and PCR amplification. The DNA isolation followed the Dilution & Storage protocol of the kit with slight modifications, and PCR amplifications of the COI barcode [43] using the mentioned primers followed the recommended PCR cycle conditions. We sequenced 51 samples from 23 countries. We additionally amplified and sequenced the fast evolving second Internal Transcribed Spacer (ITS2) of the nuclear ribosomal gene cluster of a high number of both rearing culture and field-collected samples using the primer pair ITS2a and ITS2b published by Beebe & Saul [44].

PCR products were enzymatically purified using Illustra ExoStar (GE Healthcare) and the same primers were employed for subsequent bidirectional Sanger sequencing reactions. The laboratory work was done at FMNH Luomus DNA Lab (Helsinki, Finland), and sequencing at Sequencing Service Laboratory (FIMM Genomics, UH, Helsinki, Finland). The raw sequences were edited for base-calling errors and assembled using Sequencher vs 5.0 (GeneCodes, Ann Arbor, USA).

Singapore lab. We screened 259 specimens from 13 countries representing all six continents (Supplementary Table S1) by amplifying a 313-bp fragment of COI using either direct PCR (dPCR) [45] or DNA extraction with QuickExtract [46]. The use of tagged primers allowed for sequencing these fragments on HiSeq 2500 using the pipeline described in [46, 47]. Once unique haplotypes were identified based on the 313 bp piece of COI, we carried out formal DNA extractions for specimens representing the different haplotypes. DNA was extracted using BioEr’s GenePure Plus Nucleic Acid Extractor following the protocol of the MagaBio Insect Genomic DNA Purification Kit. Extracts were subjected to PCR to amplify the 658-bp fragment of COI using LCO-HCO and a fast-evolving 270-bp fragment of 28S ribosomal RNA using the following primer pair: 28S_P03_F: 5′- TTYRGGAYACCTTYDGGAC-3′ and 28S_P03_R: 5′- GGTTTCCCCTGACTTCDACCTGATCA-3′. Sequences for the full-length DNA barcode were obtained with Sanger sequencing while the data for the 28S rDNA fragment were obtained via tagged amplicon sequencing following the same pipeline as the 313 bp COI fragment (see above).

#### Sequence analyses

We used MEGA version 7 [48] for generating a Neighbor-Joining (NJ) tree (unrooted network) [49] for all haplotypes and localities using uncorrected p-distance values, and for computing pairwise divergences among the COI barcodes. Median-Joining haplotype networks can be useful for both conveying population structures and visualizing the similarity among haplotypes. We thus generated Median-Joining haplotype networks [50] using POPART [51] for all haplotypes of field-collected samples (Fig. 2a), and separately for BSF samples from rearing cultures (Fig. 2b), with geographic origins of members of sequence clusters I-III listed in Table 1. Additionally, for the full dataset of all complete BSF barcodes we also generated a Median-Joining network (Supplementary data Fig. S1).

#### Hybridization experiments

To examine the reproductive compatibility between populations of BSF with high mitochondrial gene divergence, we carried out two batches of hybridization experiments involving virgin males and females from different cultures. The first experiment involved 10 virgin females from a culture established based on wild caught-flies in Singapore with 10 virgin males obtained from Spain of US commercial origin (uncorrected COI p-distances between Spanish and Singapore flies range between 3.7–4.0%). Virgin hybrid flies of both sexes were separated and then back-crossed to virgin flies from their parental cultures. The following reciprocal pairings were carried out: 20 Spanish ♂♂ × 20 Hybrid ♀♀; 20 Hybrid ♀♀ × 20 Singapore ♂♂. A second batch of experiments involved 311 New World ♂♂ with 310 ♀♀ from Singapore and 408 New World ♀♀ with 407 Singapore ♂♂.

## Supplementary information

**Additional file 1 : Supplementary Figure S1.** Median-Joining haplotype network of combined rearing culture and field-collected samples for the complete COI sequences. The biogeographical region of the haplotypes is color-coded, and the size of circles is proportional to the number of individuals per haplotype. The black circles represent putative un-sampled haplotypes. The branch lengths are largely proportional to the numbers of mutational steps separating the haplotypes, and the number(s) of mutational steps are indicated with hashmarks on the branches. All rearing culture samples are indicated with a black ►. The most abundant COI haplotypes are indicated with Roman numerals I-III with the geographical sources listed in Table 1.

**Additional file 2 : Supplementary Table S1.** GenBank accession numbers for each distinct haplotype and locality/culture. Specimen repository indicated for pinned fly specimens (CSCA = California State Collection of Arthropods, USA; MZH = Finnish Museum of Natural History Luomus).

**Additional file 3 : Supplementary Table S2.** List of haplotypes.

## Data Availability

The data supporting the conclusions of this article are included in the additional files of the article.

## Abbreviations

BSF: Black soldier fly
COI: Cytochrome c oxidase subunit I
HT: Haplotype
ITS2: Second Internal transcribed spacer
MY: Million years
rDNA: Ribosomal rRNA genes
WWII: World War II
