# Supplementary figures and images for: The puzzling mitochondrial phylogeography of the black soldier fly (Hermetia illucens), the commercially most important insect protein species
Source: BMC Evol Biol. 2020 May 24;20:60. doi: 10.1186/s12862-020-01627-2 (PMC7247124; doi:10.1186/s12862-020-01627-2)

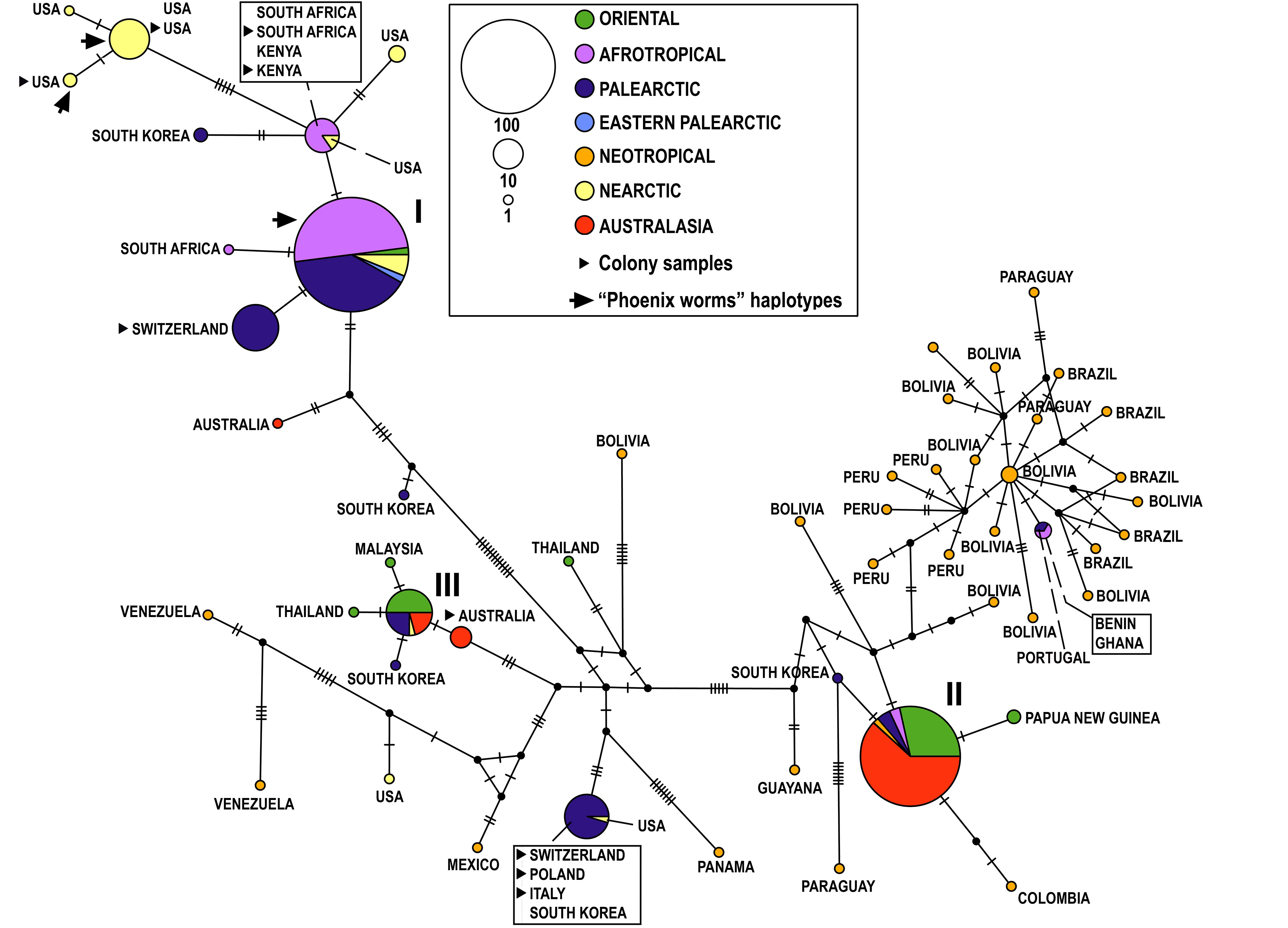

Supplement: Supplementary file 1 — Additional file 1 : Supplementary Figure S1. Median-Joining haplotype network of combined rearing culture and field-collected samples for the complete COI sequences. The biogeographical region of the haplotypes is color-coded, and the size of circles is proportional to the number of individuals per haplotype. The black circles represent putative un-sampled haplotypes. The branch lengths are largely proportional to the numbers of mutational steps separating the haplotypes, and the number(s) of mutational steps are indicated with hashmarks on the branches. All rearing culture samples are indicated with a black ►. The most abundant COI haplotypes are indicated with Roman numerals I-III with the geographical sources listed in Table 1. [file 12862_2020_1627_MOESM1_ESM.tif]
